# Association of cognitive function very early after stroke with subjective cognitive complaints after 3 months, a register-based study

**DOI:** 10.1371/journal.pone.0283667

**Published:** 2023-03-29

**Authors:** Alice Zanin, Malin Reinholdsson, Tamar Abzhandadze

**Affiliations:** 1 Faculty of Psychology, University of Padua, Padua, Italy; 2 Institute of Neuroscience and Physiology, The Sahlgrenska Academy, University of Gothenburg, Gothenburg, Sweden; 3 Department of Occupational Therapy and Physiotherapy, Sahlgrenska University Hospital, Gothenburg, Sweden; Carl von Ossietzky Universitat Oldenburg, GERMANY

## Abstract

**Objective:**

Cognitive deficits are commonly observed after stroke and have been associated with the cognitive decline and development of dementia in later stages. This study aimed to investigate whether cognition screened at acute stroke units could explain subjective cognitive complaints 3 months after stroke and evaluate how the severity of stroke and age could influence this association.

**Methods:**

In this register-based longitudinal study, data were retrieved from three Swedish registers between November 2014 and June 2019. Information on subjective cognitive complaints (SCC) was collected from the Riksstroke 3-month follow-up form, which were used to analyze the primary outcomes. Cognitive function screened using the Montreal Cognitive Assessment (MoCA) at acute stroke units was expressed as the primary independent variable.

**Results:**

Of the 1977 patients included in the study, 58% were males, the median age was 73 years, and 63% had a minor stroke. A total of 60% of patients had impaired cognition at acute stroke units (MoCA score, <26), of whom 40.3% reported at least 1 cognitive problem after 3 months. In adjusted binary regression analysis models, patients with normal cognitive function had lower odds for SCCs. This pattern was observed regardless of age and in patients with a minor stroke.

**Conclusions:**

Intact cognition early after stroke was related to decreased odds of subjective cognitive complaints at the 3-month follow-up. This study highlights the importance of both early cognitive screening after stroke and subjective cognitive complaints, which have been shown to be associated with cognitive decline. Furthermore, we suggest the importance of discussing cognitive function with patients during regular follow-up in primary care, usually 3 months after stroke.

## Introduction

Although the mortality rate of stroke is gradually decreasing, it remains a major cause of disability worldwide [1]. Therefore, much attention has been paid to the long-term residual disabilities that affect stroke survivors [2]. A major but often neglected consequence of stroke is cognitive impairment [2–4], especially in the first few weeks following the vascular event [5]. Post-stroke cognitive impairment (PSCI) is defined as the development of a new cognitive deficit within the first 3 months after stroke that persists for at least 6 months and is not related to conditions other than stroke [6]. Several cognitive domains, including memory [4,7], language [4,8,9], visuospatial [5,8,9], and executive abilities [5,8,9], can be affected by stroke and are the most common deficits observed in patients in the first few months after stroke when assessed with standardized measures. Furthermore, it has been reported that advanced age [10] and severe stroke [11] may act as further risk factors for PSCI and cognitive decline after stroke. Cognitive impairment may impede stroke recovery, and therefore early screening of cognitive function is recommended for these patients [12,13].

The Montreal Cognitive Assessment (MoCA) have been recommended as an appropriate cognitive screening test to be applied in stroke units [12,13]. MoCA has been shown to be reliable in patients with stroke during the acute and subacute phases due to its ease of use and high sensitivity in the evaluation of executive functions [14,15]. It is usually administered to patients with a mild stroke. Several studies highlighted that patients are often discharged without cognitive screening because cognitive symptoms may be mild and are not initially detected; however, they may progress slowly over time and impact the life of the individual [12]. Additionally, patients are usually followed up for 3 months after stroke by a general practitioner who may have limited knowledge regarding cognitive impairments after stroke. Thus, cognitive deficits may be overlooked during the follow-up visits, especially if patients have recovered well from the motor symptoms.

Recently, interest in subjective cognitive complaints (SCCs) has been increasing because they have been shown to affect the recovery of stroke and increase healthcare consumption [16]. SCCs are cognitive problems or difficulties perceived and reported by patients through interviews or questionnaires and differ from objective cognitive impairments, which are usually assessed using neuropsychological tests [17]. SCCs are common after a stroke, and together with PSCI and Objective cognitive impairment (OCI), they can result in dementia at later stages [18]. Based on a review by van Rijsbergen et al. [18], up to 90% of patients with stroke report cognitive problems between 1 and 54 months, especially in memory, mental speed, and orientation. Studies based on relatively small sample sizes have found conflicting results regarding association between objective and SCC for the time windows of 2 weeks, 12 months, and 4 years [19–21]. However, when considering the 3-months period, a positive association was found [22]. Early after stroke, patients both experience and show cognitive difficulties. Hence, together with early cognitive function after stroke, self-reported cognitive difficulties at follow-up may help clinicians identify patients with stroke at risk for later cognitive decline despite recovery or lack of visible impairments. Both the patients and their families may benefit from this screening since practitioners may provide useful information regarding the consequences of cognitive decline on daily life and may implement a suitable rehabilitation plan according to the patients’ complaints.

Therefore, this study aimed to investigate whether cognitive function, screened using the MoCA during the stay at acute stroke units, could explain SCCs at the 3-month follow-up after stroke and evaluate how the severity of stroke and age could influence this association.

## Material and methods

### Study sample

In this register-based longitudinal study, data were collected from three registers: Riksstroke, Väststroke, and Statistics Sweden. Riksstroke is the national quality register for stroke care in Sweden [23], while Väststroke is a local complement. Statistics Sweden contains information regarding the sociodemographic backgrounds of the participants. Patients admitted to Sahlgrenska University Hospital (SU) between November 1, 2014, and June 30, 2019, were filed in the registers. SU comprises three stroke units: one provides reperfusion treatment and has regional responsibility for thrombectomy. Data from the Väststroke register were merged with data from the Riksstroke register (acute and 3-month forms) and Statistics Sweden because the former registers lacked detailed information on patients’ sociodemographic characteristics. This merge was performed to provide extensive information about the same patient. The Riksstroke and Statistics Sweden statisticians used the patients’ personal identification numbers to link the registers. The received data file was pseudonymized. The inclusion criteria were as follows: 1) stroke diagnosis according to the International Classification of Diseases version 10 (ICD-10) criteria: I61 (intracerebral hemorrhage), I63 (cerebral infarction), and I64 (not specified stroke); 2) age ≥18 years; 3) MoCA screening at acute stroke units; and 4) completion of the Riksstroke 3-month follow-up form by patients or caregivers. Patients were excluded if SCCs data were not recorded at the 3-month follow-up.

### Procedure

Data on patients’ lives before the stroke, stroke care, and rehabilitation at stroke units were collected using the Riksstroke register. Data in Väststroke and Riksstroke’s acute form were registered by healthcare professionals at stroke units. At hospital admission, neurological function was assessed using the National Institutes of Health Stroke Scale (NIHSS) [24] by a physician or nurse, and the scores were registered in the Väststroke register. During the patients’ stay at the stroke unit (median, 7 days), cognitive function was screened by occupational therapists using the MoCA. During the study period, the MoCA was a freely available screening instrument for clinical work and official training suggested by copyright holders was not necessary. However, occupational therapists underwent training for administering the MoCA according to the SU hospital standards and regulations. At 3 months, patients replied to the Riksstroke Postal Questionnaire (Riksstroke 3-month follow-up form), and information regarding cognitive difficulties and current living arrangements was gathered. This questionnaire focused on self-perceived life after stroke, including the activities of daily living and cognition. If the form was not returned within 1 month, a reminder letter was sent, or patients were called by the nurses at stroke units.

### Outcome measures

SCCs 3 months after stroke were retrieved from the Riksstroke 3-month follow-up form. Accordingly, patients reported whether they had difficulties in speaking, understanding speech, reading, writing, counting, remembering things, concentrating, or none. Each cognitive item had two answers: yes and no. Additionally, a new variable was created by adding the number of self-reported cognitive problems ranging from 0 to 7. The variable was then dichotomized into no cognitive complaints and ≥1 cognitive complaint (acknowledging that at least one problem area was considered as having cognitive problems). The dichotomized variable was used as the primary outcome variable.

Cognitive functions at acute stroke units were screened using the MoCA. The MoCA is a one-page test comprising different cognitive domains: visuospatial abilities, memory, executive functions, attention, language, and orientation. It has a maximum score of 30, with a cutoff for normal cognitive function set at ≥26 [25].

Stroke severity at admission was assessed using the NIHSS, with a maximum score of 42, indicating a very severe stroke [24], and a cutoff score of ≤3 was chosen to indicate a minor stroke [26]; patients with a score ≥4 were grouped as having a moderate-to-severe stroke.

Sex, age (dichotomized for subgroup analysis based on age as 18–79 and ≥80 years since the age of 80 years is shown to be the median age for dementia diagnosis in Sweden [27], educational level was dichotomized, >12 years, according to the correction applied on the MoCA score [25], diagnosis, risk factors, and lengths of stay at stroke were analyzed.

### Statistical analyses

The characteristics of patients included in the study are presented with descriptive statistics; depending on the type of variable, the mean and standard deviation, median and range, or number and percentages were calculated. Comparisons between patients with impaired and intact cognition were performed using the Mann–Whitney U test for ordinal or continuous variables and Pearson’s chi-square (χ^2^) test for nominal variables. The same statistical tests were used for dropout analyses.

Spearman’s rank-order correlation analysis was used for continuous variables, whereas the phi correlation coefficient was employed for categorical variables to study the correlations between variables. The strength of correlation was interpreted as >±0.70, strong; ±0.40 to ±0.70, medium; <±0.40, weak [28].

### Regression analyses

**Fig 1:**
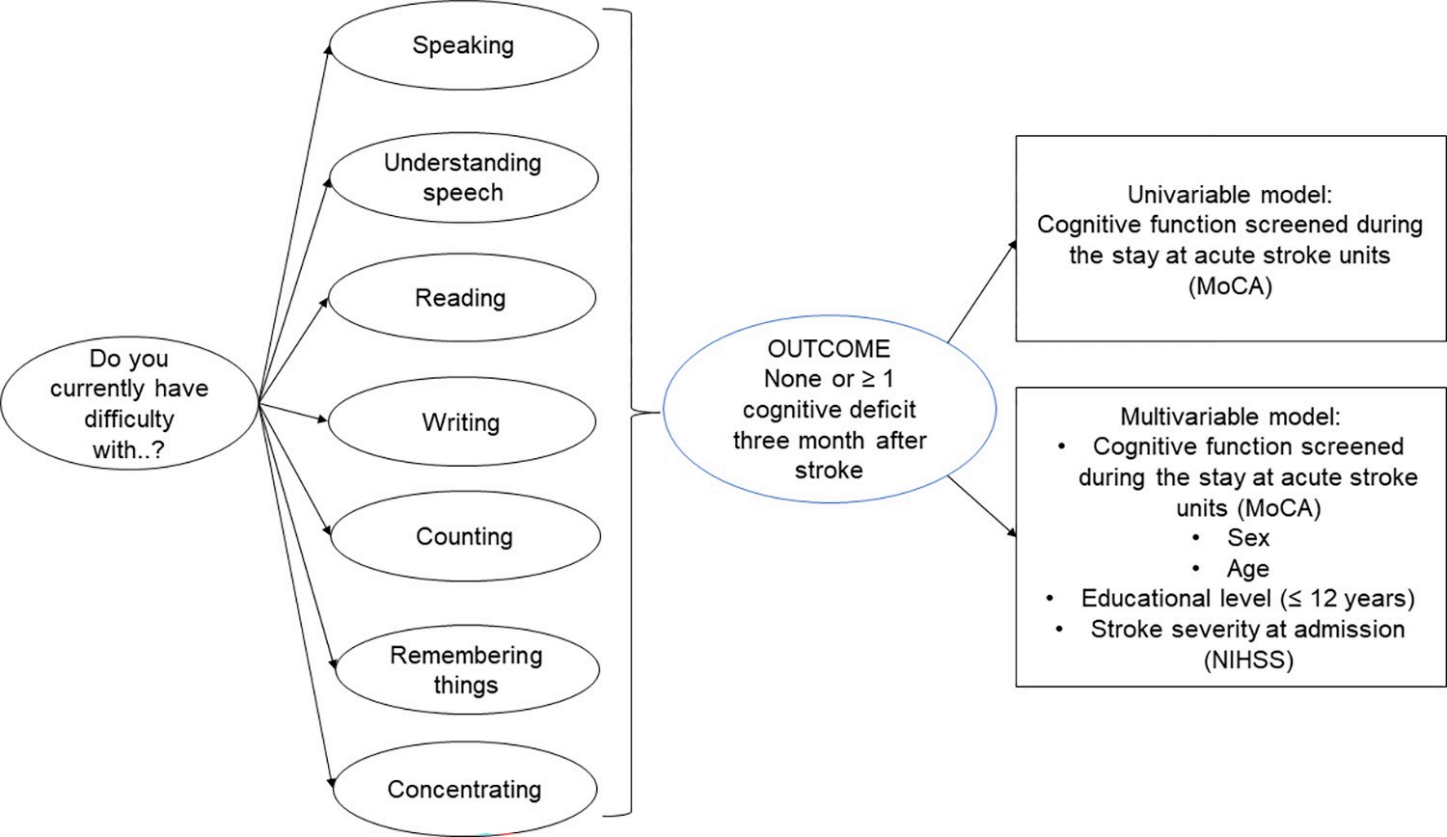
Flowchart of the binary logistic regression model. Abbreviations: MoCA, Montreal Cognitive Assessment; NIHSS, National Institutes of Health Stroke Scale.

Binary logistic regression analysis was used to study whether cognitive screening in patients at acute stroke units could explain SCCs 3 months after a stroke (“0” indicates no SCC and “1” indicates SCC with at least one cognitive dysfunction). The primary explanatory variable was the cognitive function screened at the stroke unit, and the secondary explanatory variables were sex, age, educational level, and admission stroke severity. The explanatory variables were chosen based on clinical reasoning and previous studies (Fig 1) [29].

#### Assumptions testing

Cross tables with outcome variables and categorical independent variables were investigated to test the assumption of ≥10 observations per outcome category. Multicollinearity was evaluated by correlational analysis using Spearman’s rank-order and Phi correlation coefficients.

#### Model building

Two models were built; the univariable model included only the cognitive function, and in the multivariable model, cognitive function was adjusted for other independent variables.

#### Model evaluation

The goodness of fit of the models was estimated using the Hosmer and Lemeshow test (good fit if *p* > 0.05) and the Omnibus test (*p* ≤ 0.05). The explained variance of the model was estimated using Cox and Snell’s R^2^ and Nagelkerke’s R^2^. The ability of MoCA to accurately classify patients with SCCs was estimated using the area under the curve (AUC) analysis. Odds ratios (ORs) and 95% confidence intervals (CIs) were evaluated.

Subgroup analyses were performed based on the NIHSS and age cutoffs. In the subgroup analysis related to stroke severity, two groups were created: the minor stroke group had an NIHSS score of ≤ 3, whereas the moderate-to-severe stroke group had an NIHSS score of >3. In the subgroup analysis related to age, patients were divided into those aged 18–79 years and ≥ 80 years.

Data were analyzed using IBM SPSS Statistics 26.0 (IBM SPSS Statistics for Windows, Version 26.0. Armonk, NY: IBM Corp.). The tests were two-sided at the 5% significance level.

### Ethics

The regional ethical review board in Gothenburg gave permission to use these data (amendment: 2020–01668, approved on 2020-05-14; application: 346–16, approved on 2016-05-04). According to the Swedish Data Protection Authority, quality register data represent an exception to obtaining patients’ written informed consent; therefore, written or oral informed consent was not collected in this study. Moreover, data from medical charts can be collected for clinical purposes and quality control according to the Personal Data Act (Swedish Law #1998:204, issued on 1998-04-29) without written informed consent.

## Results

### Patient characteristics

**Fig 2:**
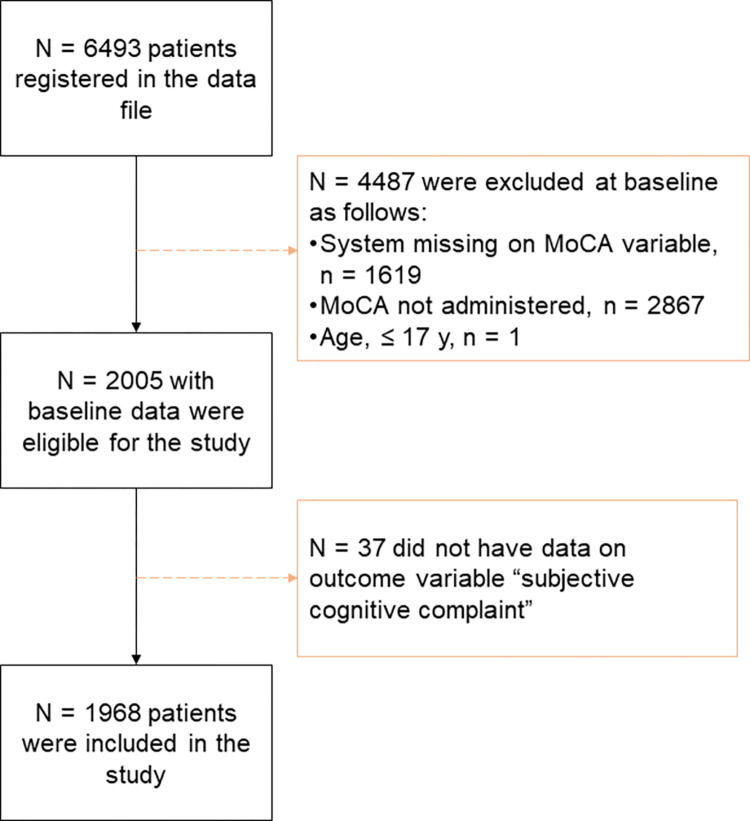
Flowchart of the study participants. Abbreviations: MoCA, Montreal Cognitive Assessment.

A total of 1977 patients were included in this study according to the inclusion criteria and were admitted to stroke units between November 2014 and June 2019 (Fig 2).

The dropout analyses revealed significant differences in sex, age, and stroke severity at admission (*p* < 0.001) between the excluded and included patients (Table 1).

**Table 1:** Comparison between patients included in the study sample and excluded patients.

Characteristics	Excluded patients N = 4514	Included patients N = 1977	P-value
Sex, n (%):			**<0.001**
Male	2255 (50)	1157 (58)	
Female	2259 (50)	820 (42)	
Age			**<0.001**
Mean (SD)	74.9 (13.9)	71.5 (13.3)	
Median (IQR)	77 (18 [18–103])	73 (17 [20–99])	
Stroke severity (NIHSS)			**<0.001**
Mean (SD)	7.0 (7.4)	2.3 (3.9)	
Median (range [IQR])	4 (11 [0–36])	1 (3 [0–30])	
Having motor impairment in upper extremity*, n (%):			
Right	671 (21)	98 (7)	**<0.001**
Left	703 (22)	216 (12)	**<0.001**
Have problems with understanding*, n (%):	574 (18)	58 (3)	**<0.001**
Have problems with communication / language*, n (%)	1200 (39)	265 (15)	**<0.001**
Having dysarthria*, n (%)	1361 (45)	417 (23)	**<0.001**

Statistics: Bold text indicates statistically significant differences (p < 0.05), Chi-squared test was used for binary variables and Mann-Whitney U test was used for continuous variables.

Abbreviations: SD, standard deviation; IQR, interquartile range, NIHSS, National Institutes of Health Stroke Scale.

*Neurological domains from NIHSS.

In the study sample, the patients primarily had minor strokes (median NIHSS score, 1), the median age was 73 years, 58% were male, and 60% had cognitive impairment (MoCA <26), (Table 2).

**Table 2:** Characteristics of patients included in the study—Baseline data. Between-group analyses of patients with impaired (MoCA <26) and intact (MoCA ≥26) cognition at acute stroke units.

Characteristics	Total sample (n = 1977)	Normal cognition, MoCA ≥26 (n = 792)	Impaired cognition, MoCA <26 (n = 1185)	P-value
Sex, n (%):				**<0.001**
* *Male	1157 (58)	498 (63)	659 (56)	
* *Female	820 (42)	294 (37)	526 (44)	
Age, years				**<0.001**
* *Mean (SD)	71.5 (13.3)	66.9(13.7)	74.6 (12.1)	
* *Median (IQR [range])	73 (17 [20–99])	69 (18 [20–92])	76 (14 [20–99])	
Educational level, n (%):				**<0.001**
* *≤ 12 years	972 (49)	325 (41)	647 (55)	
* *> 12 years	1005 (51)	467 (59)	538 (45)	
Diagnosis, n (%):				**0.001**
* *Primary cerebral hemorrhage	152 (7.7)	42 (5)	110 (9)	
* *Ischemic stroke	1825 (92)	750 (95)	1075 (91)	
Risk factors, yes, n (%):				
* *Previous stroke	237 (12)	69 (9)	168 (14)	<0.001
* *Previous TIA	149 (8)	44 (6)	105 (9)	**0.005**
* *Atrial fibrillation	417 (21)	117 (15)	300 (25)	**<0.001**
* *Diabetes	374 (18)	109 (14)	238 (20)	**<0.001**
* *Smoking ≥ 1 cigarette per day or stopped in the last 3 months	276 (15)	103 (14)	173 (16)	0.15
Length of stay at stroke unit (days):				**<0.001**
* *Mean (SD)	10.4 (9.3)	7.9 (6)	12.1 (9.6)	
* *Median (IQR [range])	7 (8 [0–100])	6 (4 [0–100])	10 (11 [0–100])	
Admission stroke severity, NIHSS				**<0.001**
* *Mean (SD)	2.5 (3.9)	1.5 (2.7)	3.2 (4.4)	
* *Median (IQR [range])	1 (3 [0–30])	0 (2 [0–24])	2 (4 [0–30])	
Cognitive function, MoCA				**<0.001**
* *Mean (SD)	23.4 (4.7)	27.5 (1.3)	20.1 (4.2)	
* *Median (IQR [range])	25 (6 [1–30)	27 (2 [26–30])	22 (5 [1–25])	

Bold text indicates statistically significant differences (p < 0.05) between patients with impaired and normal cognition in the stroke units; Chi-squared test was used for binary variables and Mann-Whitney U test was used for continuous variables.

Abbreviations: SD, standard deviation; TIA, transient ischemic attack; NIHSS, National Institutes of Health Stroke Scale; MoCA, Montreal Cognitive Assessment.

Variables with missing values, n (% of total study sample): Educational level, 68 (3.5); previous stroke, 6 (0.3); previous TIA, 20 (1.0); atrial fibrillation, 4 (0.2); diabetes, 2 (0.1); smoking, 181 (9.2); length of stay at stroke unit, 107 (5.4); NIHSS, 363 (18.4).

**Fig 3:**
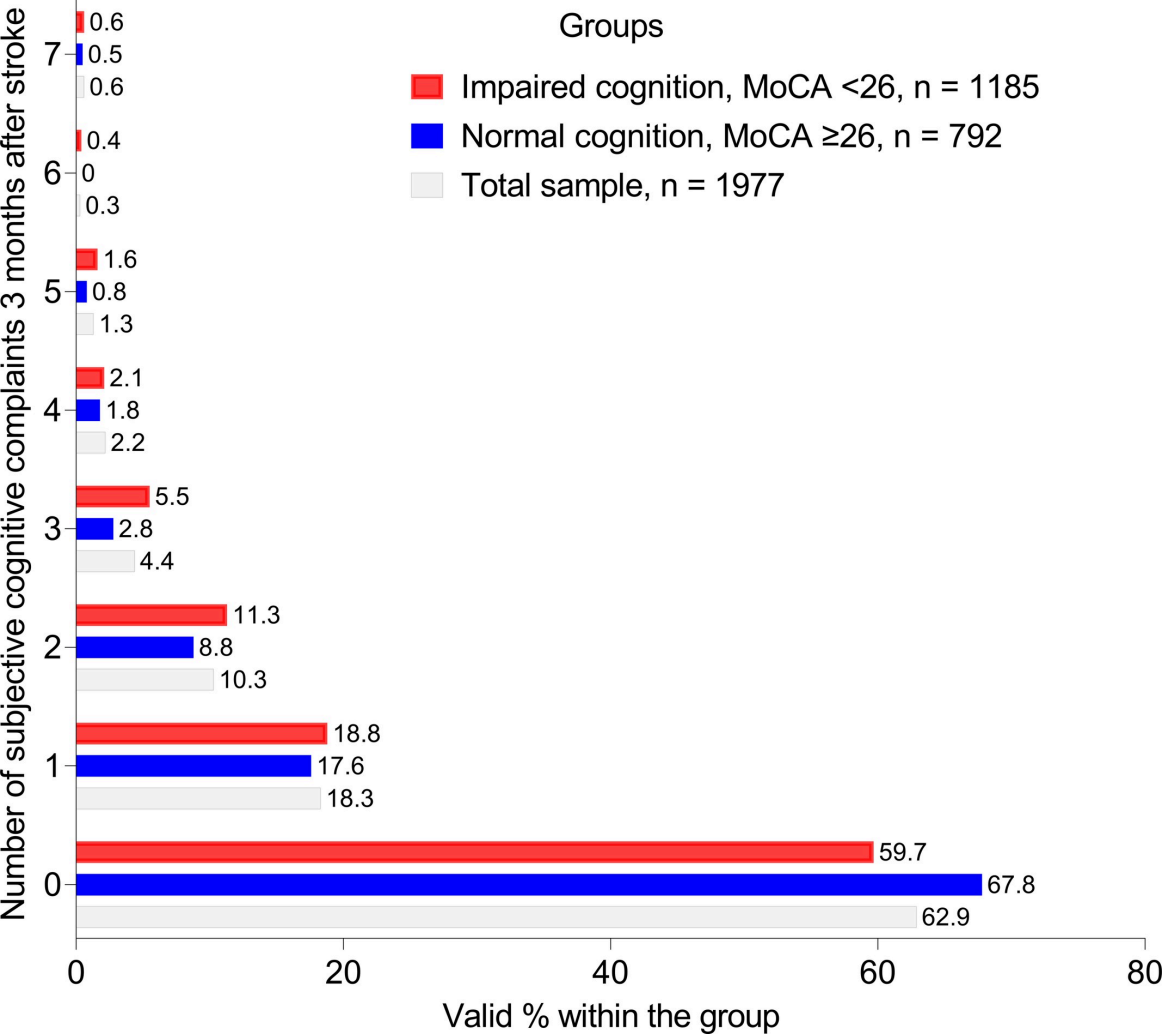
Number of subjective cognitive complaints in the total sample as well stratified based on the cognitive function (screened with the Montreal Cognitive Assessment [MoCA]) at baseline.

After 3 months, 733 (37%) patients reported at least one cognitive deficit, whereas 1244 (63%) did not report any deficits, Fig 3.

After 3 months, 21% of the patients reported problems with memory function and 2% reported problems with understanding, (Table 3).

**Table 3:** S*ubjective cognitive complaints* 3 months after stroke in the total sample as well as stratified based on cognitive function at baseline.

Characteristics	Total study sample (N = 1977)	Normal cognition, MoCA ≥26 (N = 789)	Impaired cognition, MoCA <26 (N = 1180)	P-value
***Subjective cognitive complaints*, 3 months**				**< 0.001**
Mean (SD)	0.7 (1.2)	0.6 (1.1)	0.8 (1.3)	
Median (IQR [range])	0 (1 (0–7])	0 (1 [0–7])	0 (1 [0–7])	
**Types of *subjective cognitive complaints* at 3 months**				
* *Problem in speaking, yes, n (%)	156 (8)	45 (6)	111 (9)	**0.003**
* *Problem in understanding speech, yes, n (%)	41 (2)	10 (1)	31 (3)	**0.04**
* *Problem in reading, yes, n (%)	168 (9)	51 (6)	117 (10)	**0.007**
* *Problem in writing, yes, n (%)	202 (10)	54 (7)	148 (13)	**< 0.001**
* *Problem in counting, yes, n (%)	81 (4)	18 (2)	63 (5)	**<0.001**
* *Problem in remembering things, yes, n (%)	420 (21)	138 (17)	282 (24)	**<0.001**
* *Problem in concentrating, yes, n (%)	351 (18)	143 (18)	208 (18)	0.77

Abbreviations: MoCA, Montreal Cognitive Assessment, administered during the stay at acute stroke units. Statistics: Bold text indicates statistically significant differences (p < 0.05) between patients with impaired and normal cognition in the stroke units; Chi-squared test was used for binary variables and Mann-Whitney U test was used for a continuous variable.

### Relationship between cognitive functions screened using MoCA and SCCs 3 months after stroke

**Fig 4:**
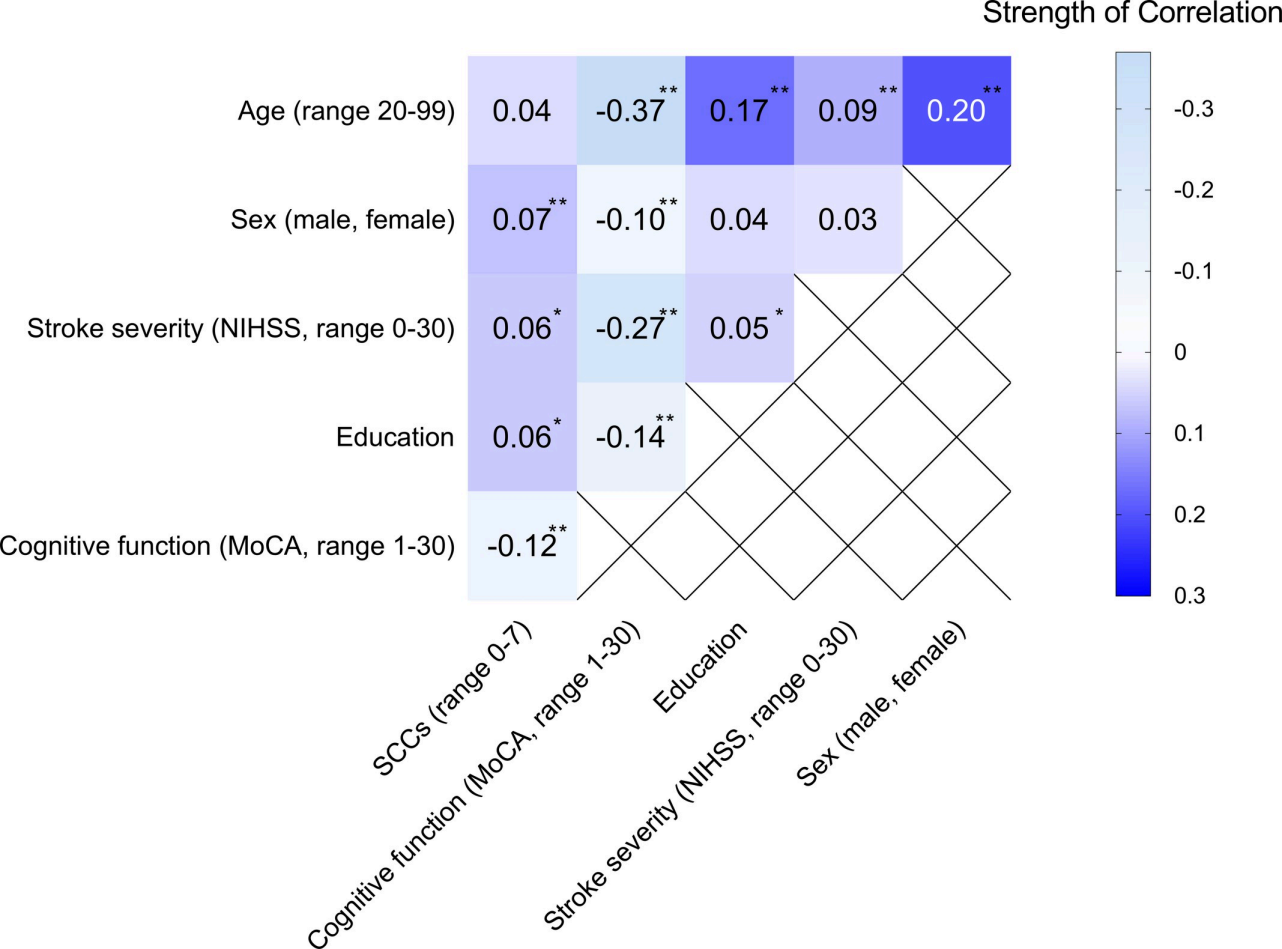
Correlational analysis between cognitive function screened at stroke units (Montreal Cognitive Assessment [MoCA]), age, sex, education, stroke severity at admission to the hospital (the National Institutes of Health Stroke Scale [NIHSS]), and subjective cognitive complaints (SCCs) three months after stroke. Statistics: Spearman’s rank-order correlation coefficient for continuous variables, Phi correlation coefficient for nominal variables. ** p < 0.01; *p < 0.05.

Correlational analysis showed a significantly weak negative correlation between the cognitive function screened using MoCA and SCCs 3 months after stroke (Fig 4).

**Fig 5:**
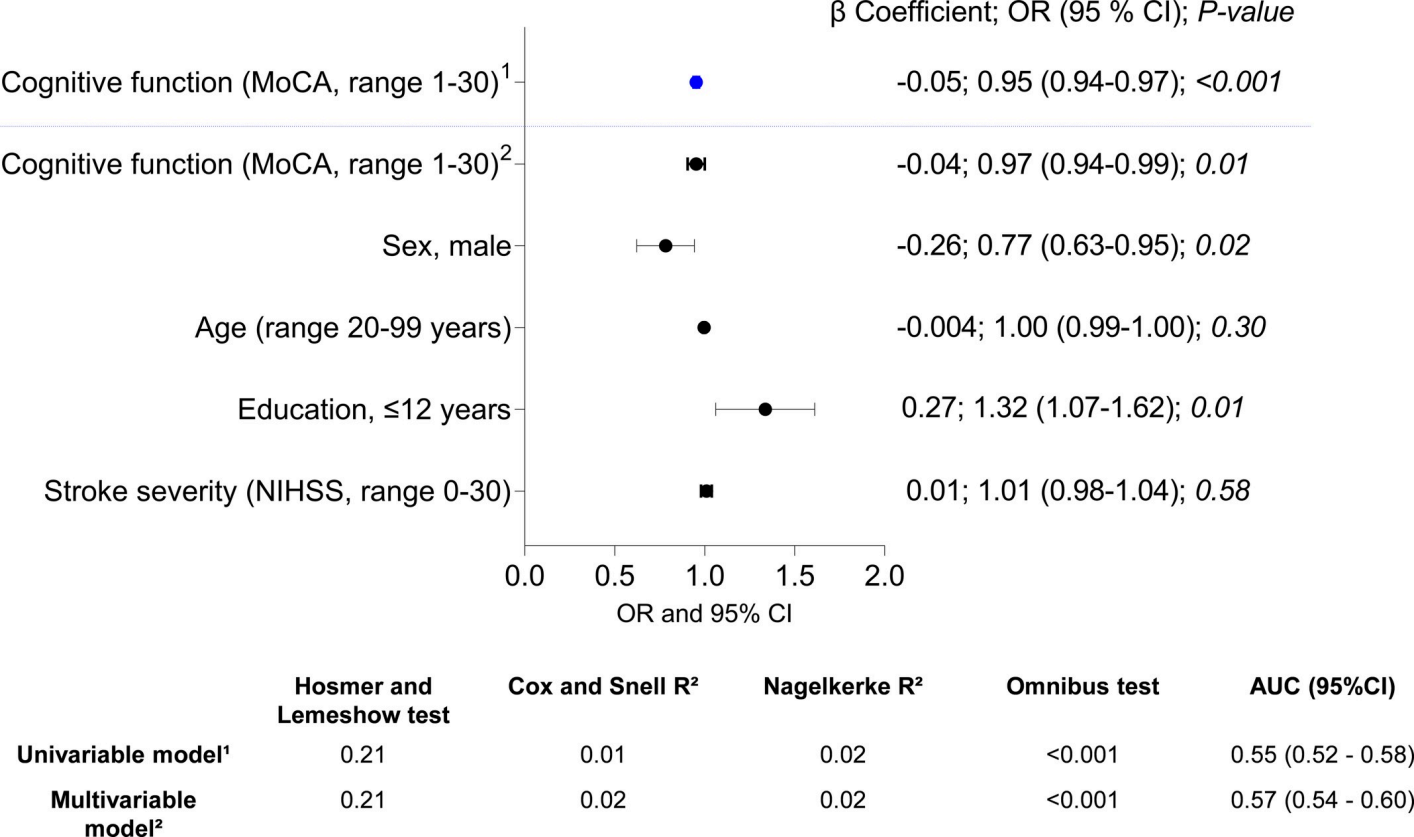
Forest plot with the results of binary logistic regression analyses showing the explanatory value of independent variables for subjective cognitive complaints (SCCs) 3 months after stroke. MoCA, Montreal Cognitive Assessment; NIHSS, National Institutes of Health Stroke Scale; AUC, area under the curve; CI, confidence interval; ORs, odds ratios.

In a univariable model, patients with intact cognition had lower odds of reporting problems in at least one cognitive function 3 months after stroke. When the cognitive function was adjusted for other variables, the odds ratio decreased (Fig 5).

### Subgroup analyses

Intact cognition could significantly explain the lower odds of SCCs at 3 months of follow-up in patients with minor stroke (NIHSS score ≤3), Table 4. In the subgroup analysis related to age, intact cognition was a significant explanatory variable for both age groups (<80 years and ≥80 years); regardless of age, patients with intact cognition had significantly lower odds of reporting SCCs at 3 months after stroke, Table 4.

**Table 4:** The results of binary logistic regression analyses showing the explanatory value of cognitive function screened early after stroke for subjective cognitive compliant after 3 months.

Sub-groups^#^	Explanatory variables	β coefficient	OR (95% CI)	P-value	Hosmer & Lemeshow	Cox & Snell R^2^	Nagelkerke R^2^	Omnibus test	AUC (95% CI)
NIHSS ≤3 (N = 1253)	Cognitive function (MoCA, range 1–30)	-0.04	0.96 (0.94–0.99)	**0.006**	0.39	0.006	0.01	0.006	0.54(0.51–0.58)
	Cognitive function (MoCA, range 1–30)	-0.03	0.97 (0.94–0.99)	**0.02**	0.89	0.02	0.02	<0.001	0.58(0.54–0.61)
	Sex, male	-0.37	0.69 (0.54–0.88)	**0.002**					
	Age, years (range 20–98)	-0.00	1.00 (0.99–1.01)	0.47					
	Educational level, ≤12 years	0.23	1.25 (0.99–1.59)	0.06					
NIHSS ≥4 (N = 361)	Cognitive function (MoCA, range 2–30)	-0.04	0.97 (0.93–1.01)	0.09	0.54	0.008	0.01	0.09	0.56(0.49–0.62)
	Cognitive function (MoCA, range 2–30)	-0.04	0.96 (0.92–1.00)	0.08	0.56	0.02	0.03	0.12	0.58(0.51–0.64)
	Sex, male	0.10	1.10 (0.72–1.72)	0.64					
	Age, years (range 24–99)	-0.01	0.99 (0.97–1.01)	0.31					
	Educational level, ≤12 years	0.44	1.55 (0.99–2.43)	0.05					
Age <80 years (N = 1159)	Cognitive function (MoCA, range 5–30)	-0.04	0.96 (0.93–0.98)	**<0.001**	0.55	0.01	0.01	<0.001	0.55(0.52–0.58)
	Cognitive function (MoCA, range 5–30)	-0.02	0.98 (0.95–1.01)	0.16	0.43	0.02	0.02	0.02	0.57(0.54–0.60)
	Sex, male	-0.36	0.69 (0.54–0.89)	**0.004**					
	Educational level, ≤12 years	0.28	1.32 (1.04–1.69)	**0.03**					
	Stroke severity (NIHSS, range 0–30)	0.01	1.01 (0.98–1.04)	0.64					
Age, ≥80 years (N = 455)	Cognitive function (MoCA, range 1–30)	-0.06	0.94 (0.91–0.97)	**<0.001**	0.06	0.02	0.03	<0.001	0.60(0.55–0.64)
	Cognitive function (MoCA, range 1–30)	-0.05	0.95 (0.91–0.99)	**0.01**	0.79	0.02	0.03	0.06	0.58(0.53–0.64)
	Sex, male	0.02	1.02 (0.69–1.50)	0.93					
	Educational level, ≤12 years	0.24	1.27 (0.86–1.89)	0.23					
	Stroke severity (NIHSS, range 0–24)	0.01	1.01 (0.96–1.06)	0.70					

The bold text indicates statistically significant results (*p* < 0.05). Abbreviations: OR, odds ratio; CI, confidence interval; AUC, area under the curve; MoCA, Montreal Cognitive Assessment; NIHSS, the National Institutes of Health Stroke Scale.

^#^ *Descriptive characteristics of the sub-groups*

NIHSS ≤3: Mean (SD), 0.9 (1.0); Median (min–max/range), 0 (0–3/3).

NIHSS ≥4: Mean (SD), 8.1 (4.9); Median (min–max/range), 6 (4–30/26).

Age, <80 years: Mean (SD), 65.8 (11.4); Median (min–max / range), 69.0 (20–79/59).

Age, ≥80 years: Mean (SD), 86.5 (4.0); Median (min–max/range), 85 (80–99/19).

## Discussion

The primary finding of this study was that cognitive function screened using MoCA during the stay at acute stroke units was significantly associated with SCCs reported by patients 3 months after a stroke event. This result may be helpful in the clinical setting for the early identification of patients with persistent cognitive difficulties after discharge from stroke units. Previous studies have reported the ability of MoCA to predict cognitive deficits, assessed through standardized measures in the first few months following a stroke [14,30,31]. However, to our knowledge, this is the first study to investigate the independent association between cognitive function screened at acute stroke units and self-reported cognitive problems.

In the present study, 37% of patients have reported SCCs in at least one cognitive domain 3 months after stroke, with most patients experiencing problems with memory (21.1%). Previous studies investigating cognitive function 3 months after stroke reported 35.2% [4] and 42.7% [32] of patients with cognitive difficulties. Therefore, our study may be helpful in the validation of the Riksstroke questionnaire. Since patients with impaired cognition at acute stroke units are more likely to report cognitive problems after 3 months, this study suggests that items reported on Riksstroke can adequately represent the most common cognitive difficulties that affect patients after stroke. Indeed, the most reported cognitive problem 3 months after stroke was memory in this study, and this result is in line with previous studies [4,7,18]. These findings may be limited by the lack of an objective measure that could prove the presence of actual cognitive deficits, as self-reports may be hindered by patients’ lack of insight or underestimation of their problems [33]. However, previous studies have reported contrasting evidence regarding the association between objective cognitive impairment and SCCs. One study from 2017 investigated whether these two constructs were related 3 months after stroke; they found a significant association between OCI and SCCs [17].

According to subgroup analyses, cognitive function screened at acute stroke units was associated with SCCs 3 months post-stroke, regardless of the patients’ age and in those with a minor stroke. Additionally, compared with 35.5% of patients with a minor stroke, 41% of patients with moderate-to-severe stroke reported at least one SCC 3 months after stroke. One possible explanation for the lack of association between cognition screened at acute stroke units and SCCs 3 months after stroke could be the high variability among the reported SCCs in patients with a severe stroke. Indeed, patients with severe neurological impairments may be unaware of their problems. Furthermore, patients with moderate-to-very severe stroke were grouped together in our study, although they showed varying severity of symptoms. Patients with a moderate stroke could still have an intact awareness of their condition in the first few months after a stroke; however, when grouped with those with a more severe stroke, the association between cognition of patients at acute stroke units and SCCs reported after 3 months could be lost.

In patients with a moderate-to-severe stroke but not in those with a minor stroke, the educational level was a significant explanatory variable for SCCs, with more years of education being associated with lower odds of reporting SCCs 3 months after a stroke. This result is in line with previous studies that reported a negative association between the educational level and cognitive decline after stroke [34,35]. Education is part of the broader concept of cognitive reserve, which is the capacity of the brain to overcome an injury based on the engagement in challenging activities an individual has during their lifespan [36]. From the results of this study, we can conclude that higher cognitive reserve plays a protective role against moderate and severe stroke, leading to lower odds for SCCs 3 months after the vascular event. However, another study reported that this association was observed in patients with a low degree of brain pathology (defined as lacunar lesion volume) [37], whereas our results reported a lower degree of brain pathology in patients with a severe stroke as assessed by the NIHSS. Future studies to better define the concept of brain pathology to allow for comparisons between different publications are needed.

## Strength and limitations

This study has several strengths and limitations. The first strength lies in the sample size, which was large enough to ensure the reliability of the results. Significant differences were found between excluded and included patients in age, sex, and NIHSS scores, suggesting that our results may not be generalizable to the whole world population with stroke, but may be comparable to the Swedish population; our sample had a median age of 73 years, whereas the median age of stroke onset in Swedish patients is 75 years. Moreover, our sample primarily presented with minor strokes, similar to the Swedish stroke population [38]. Limitations can be found in the MoCA, the cognitive screening tool employed; the chosen cutoff for normal cognition was set at 26 according to Nasreddine et al. [25]; however, it could be argued that for patients with stroke, it may be too high, especially since screening was performed during the patients’ stay at the stroke unit, in which many confounders may influence the MoCA score (such as delirium [39], depression [40] or apathy [41]. However, there is still no clear consensus on a reliable cutoff to identify vascular cognitive impairment using the MoCA, with studies reporting cutoffs ranging from 24/25 [42] to 21/22 [14]. Future studies may replicate the methodology of the current study by choosing a different MoCA cut-off value. Moreover, although the MoCA is a feasible tool for cognition, not all patients undergo screening. There are several barriers to cognitive screening at acute stroke units, that can be related to patient characteristics as well as stuff- and organization-related factors [43], thus the high proportion of the patients who did not have cognitive screening performed at stroke units. Another limitation lies in the administration of the MoCA, as it was carried out by different assessors with different patients. Information bias might have influenced the total score of the screening tool because of different assumptions that the assessors may have made regarding patients’ performance. Additionally, our study may have been subjected to selection bias since the MoCA is not a suitable cognitive screening tool in patients with a severe stroke.

The patients receive information on their MoCA results after screening, but it is unclear how much patients remember from this, particularly those patients who have impaired cognitive function. The risk of recall bias is unknown but is likely to be low. The study outcome was SCC. However, the SCC may be subject to bias, for instance, due to memory problems or lack of self-awareness. However, it is important to take such complaints seriously, as SCC can be associated with future cognitive functioning. Moreover, SCC can be related to the psychological characteristics and consequences of stroke. Therefore, SCC should be completed with the information collected via objective cognitive assessments.

## Conclusions

Cognitive function screened at acute stroke units was associated with SCCs 3 months after stroke. This tendency remained regardless of age and those with a minor stroke, whereas, for patients with a more severe stroke, educational level was a protective factor for decreased SCCs. The primary result of this study is further evidence of the need to screen patients with stroke for cognitive deficits in acute or subacute phases to identify patients at risk of cognitive decline. Furthermore, we conclude that the SCC is a reliable measure to detect the presence of cognitive deficits in these patients. SSCs should be considered during follow-up visits as they have the potential to track the progression of recovery.
